# Nociception Control of Bilateral Single-Shot Erector Spinae Plane Block Compared to No Block in Open Heart Surgery—A Post Hoc Analysis of the NESP Randomized Controlled Clinical Trial

**DOI:** 10.3390/medicina59020265

**Published:** 2023-01-30

**Authors:** Cosmin Balan, Dana R. Tomescu, Serban I. Bubenek-Turconi

**Affiliations:** 1 Cardiac Anesthesiology and Intensive Care Department I, Prof. Dr. C.C. Iliescu Emergency Institute for Cardiovascular Diseases, 022328 Bucharest, Romania; 2 Anesthesiology and Intensive Care Department, Carol Davila University of Medicine and Pharmacy, 050474 Bucharest, Romania; 3 3rd Department of Anesthesiology and Intensive Care, Fundeni Clinical Institute, 022328 Bucharest, Romania

**Keywords:** erector spinae plane block, open heart surgery, nociception monitoring, nociception level index, analgesia

## Abstract

*Background and Objectives:* The erector spinae plane block (ESPB) is an analgesic adjunct demonstrated to reduce intraoperative opioid consumption within a Nociception Level (NOL) index-directed anesthetic protocol. We aimed to examine the ESPB effect on the quality of intraoperative nociception control evaluated with the NOL index. *Materials and Methods:* This is a post hoc analysis of the NESP (Nociception Level Index-Directed Erector Spinae Plane Block in Open Heart Surgery) randomized controlled trial. Eighty-five adult patients undergoing on-pump cardiac surgery were allocated to group 1 (Control, *n* = 43) and group 2 (ESPB, *n* = 42). Both groups received general anesthesia. Preoperatively, group 2 received bilateral single-shot ESPB (1.5 mg/kg/side 0.5% ropivacaine mixed with dexamethasone 8 mg/20 mL). Until cardiopulmonary bypass (CPB) was initiated, fentanyl administration was individualized using the NOL index. The NOL index was compared at five time points: pre-incision (T1), post-incision (T2), pre-sternotomy (T3), post-sternotomy (T4), and pre-CPB (T5). On a scale from 0 (no nociception) to 100 (extreme nociception), a NOL index > 25 was considered an inadequate response to noxious stimuli. *Results:* The average NOL index across the five time points in group 2 to group 1 was 12.78 ± 0.8 vs. 24.18 ± 0.79 (*p* < 0.001). The NOL index was significantly lower in the ESPB-to-Control group at T2 (12.95 ± 1.49 vs. 35.97 ± 1.47), T3 (13.28 ± 1.49 vs. 24.44 ± 1.47), and T4 (15.52 ± 1.49 vs. 34.39 ± 1.47) (*p* < 0.001) but not at T1 and T5. Compared to controls, significantly fewer ESPB patients reached a NOL index > 25 at T2 (4.7% vs. 79%), T3 (0% vs. 37.2%), and T4 (7.1% vs. 79%) (*p* < 0.001). *Conclusions:* The addition of bilateral single-shot ESPB to general anesthesia during cardiac surgery improved the quality of intraoperative nociception control according to a NOL index-based evaluation.

## 1. Introduction

Cardiac surgery entails risks and complications uncommonly met in noncardiac surgery. Postoperative outcomes and quality of life depend on several factors, of which the patient’s risk and the type and quality of the surgical procedure play a significant role [1]. Novel programs of enhanced recovery after surgery (ERAS) require alternative options to further reduce pain, morbidity, hospitalization, and healthcare costs.

Pain management has evolved in open heart surgery to minimize the risks rather than to maximize the analgesic potential [2]. Consequently, an excellent risk–benefit ratio has sparked interest in exploring the versatility of ultrasound (US)-guided chest wall fascial plane blocks [3,4]. Lately, bilateral erector spinae plane block (ESPB) has emerged as an efficacious multimodal adjunct to achieving ERAS endpoints after midline sternotomy [5,6,7,8].

A thoracic ESPB is performed by depositing local anesthetic (LA) underneath the erector spinae muscle and superficially to the T5 transverse process, resulting in multilevel ipsilateral chest wall analgesia [9]. However, preemptive ESPB does not eliminate but only spares the intraoperative opioid consumption, an effect with duration and intensity regulated by several factors, including the block technique, the choice of LA and adjuvants, and the patient’s sensory mapping [10]. Conveniently, objective real-time nociception monitoring might help avoid intraoperative opioid overdosing or underdosing, both known to contribute to a protracted postoperative course [11].

One such monitor, the PMD-200TM (Medasense Biometrics Ltd., Ramat Gan, Israel), nonlinearly combines photoplethysmography, skin conductance, peripheral temperature, and accelerometry to derive the Nociception Level (NOL) index, a multidimensional measure of nociception–antinociception (NAN) balance [12]. The machine learning algorithm continuously integrates data to provide personalized NAN profiling. The NOL index outperformed clinical signs of stress-induced sympathetic nervous activation (i.e., heart rate, blood pressure) [13] and remained discriminative when regional and general anesthesia were combined [14]. The reported effects on intraoperative opioid consumption were conflicting [15,16], but postoperative pain scores improved consistently with NOL index protocols, presumably due to the more objective timing of opioid administration [16,17].

We recently published the results of the NESP (Nociception Level Index-Directed Erector Spinae Plane Block in Open Heart Surgery) randomized controlled clinical trial [18]. In the NESP trial, we compared NOL index-directed general anesthesia (NDGA) to NDGA combined with preemptive bilateral single-shot ESPB. To the authors’ knowledge, this was the first study to apply the NOL index in open heart surgery intraoperatively. Fentanyl administration was individualized in both groups using NOL index monitoring from the induction of anesthesia until the initiation of a cardiopulmonary bypass (CPB). Following CPB initiation, fentanyl administration was based on hemodynamic indices combined with clinical reasoning. The ESPB group had reduced intraoperative fentanyl consumption and 48 h postoperative morphine consumption, improved postoperative pain scores up to 48 h after extubation, and shorter time to extubation and liberation from vasopressors. In this post hoc analysis, the authors sought to compare the study groups by the quality of intraoperative nociception control assessed with the NOL index from the induction of anesthesia until CPB initiation.

## 2. Materials and Methods

### 2.1. Study Design and Patient Enrollment

The original NESP trial was registered with ClinicalTrials.gov (Identifier: NCT04338984) and was approved by the Institutional Review Board for Biomedical Research of Prof. Dr. C.C. Iliescu Emergency Institute for Cardiovascular Diseases, Bucharest, Romania (2019.07.26/18750) [18].

Adult patients from a single tertiary center undergoing on-pump cardiac surgery were enrolled from December 2019 to May 2021 if they met the eligibility criteria: age 18 to 75 years, elective surgery, and sinus rhythm. All the patients provided written consent. The exclusion criteria included: allergy to drugs used in the study; body mass index >35; bleeding disorder or abnormal coagulation profile; emergency or redo surgery; American Society of Anesthesiologists class >3; preoperative cardiocirculatory support; and a left ventricular ejection fraction <30%. After open-label block randomization, patients were assigned (1:1) to either NDGA (group 1, Control) or bilateral single-shot ESPB followed by NDGA (group 2, ESPB).

For this post hoc analysis of the NESP trial, we included all the patients from the original trial and two more ESPB patients excluded from the initial analysis due to immediate postoperative complications.

### 2.2. Anesthesia

Detailed methods were previously reported [18]. In brief, all the patients received standard cardiac anesthesia monitoring. A bispectral index (BIS) forehead sensor was applied to ensure adequate hypnosis during anesthesia (i.e., BIS values between 40 and 60). In addition, the PMD-200TM (Medasense Biometrics Ltd., Ramat Gan, Israel) monitor was connected to the patient by the NOL index probe placed on the middle finger, contralaterally to the arterial line. Calibration of the NOL monitor took place during a stress-free period.

Before the induction of anesthesia, a single investigator performed all the blocks (i.e., bilateral single-shot US-guided ESPB) with the patient in the sitting position, using an in-plane, caudocranial needle insertion in the fascial plane between the erector spinae muscle and the T5-T6 transverse process. Ropivacaine 0.5% 1.5 mg/kg and dexamethasone 8 mg/20 mL were administered on each side after correct positioning was confirmed by hydrolocation.

The induction of anesthesia was standardized, consisting of propofol 1.5 mg/kg, fentanyl 5 µg/kg, and rocuronium 0.6 mg/kg, followed by protective mechanical ventilation, antimicrobial prophylaxis, and stress ulcer prophylaxis.

**Figure 1:**
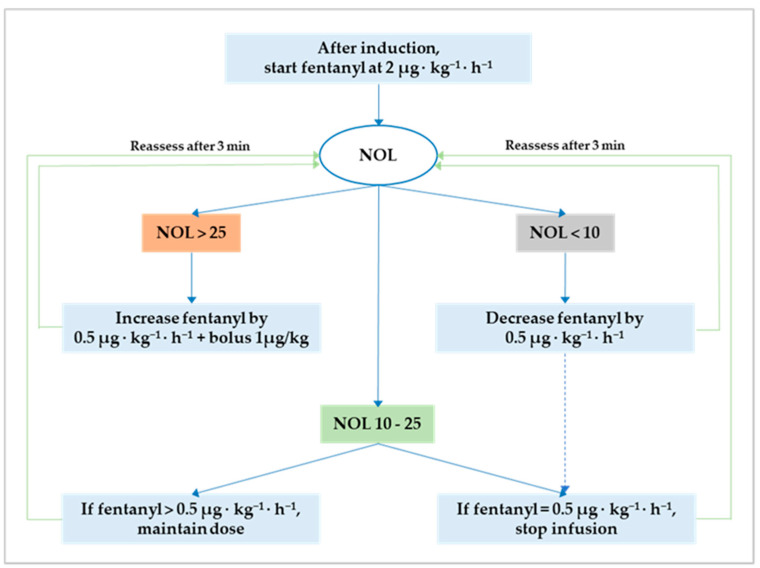
Flowchart for NOL index-based fentanyl administration. The optimum nociception–antinociception balance is a NOL index between 10 and 25. Outside this range, fentanyl should be either decreased to avoid overdosing (i.e., NOL index < 10) or increased to prevent underdosing and nociception (i.e., NOL index > 25). NOL, nociception level.

Anesthesia was maintained either with sevoflurane before and after CPB or propofol during CPB. The NOL index was continuously monitored and available for decision-making only from the induction of anesthesia until CPB initiation. This restriction is inherent to the photoplethysmographic nature of the input signal that renders the output (i.e., the NOL index) ineffective during CPB and potentially faulty after CPB discontinuation due to temporary cardiac pacing. In both groups, intraoperative fentanyl administration followed a standard protocol to attain optimum NAN balance defined as a NOL index between 10 and 25 (i.e., 10 ≤ target NOL index ≤ 25) on a scale from 0 (i.e., absence of nociception) to 100 (i.e., extreme nociception). The original NAN protocol is reiterated herein [18]: (1) after induction, start fentanyl at 2 µg·kg^−1^·h^−1^ in both groups; (2) if the NOL index is >25 for more than 60 s, increase the infusion by 0.5 µg·kg^−1^·h^−1^ and bolus 1 µg/kg; (3) if the NOL index is <10 for more than 60 s, decrease the infusion by 0.5 µg·kg^−1^·h^−1^; (4) after a dose change, allow an observation window of three minutes; and (5) stop the fentanyl infusion when it reaches 0.5 µg·kg^−1^·h^−1^ with the NOL index ≤ 25 for more than ten minutes (Figure 1). After CPB initiation, fentanyl administration was left to the discretion of the attending anesthetist, who had to pursue hemodynamic stability within 15% of the mean arterial pressure recorded during optimum NAN balance.

Postoperatively, all the patients were admitted to the intensive care unit and extubated when a minimum set of weaning criteria was met. Analgesia included intravenous paracetamol 1 g every 6 h and morphine 0.03 mg/kg to achieve numeric rating scale (NRS) scores lower than 4 (i.e., NRS ≤ 3).

### 2.3. Outcomes

Study groups were compared by the quality of intraoperative nociception control assessed with the NOL index. Accordingly, the NOL index was sampled at five distinct time points: pre-incision, post-incision, pre-sternotomy, post-sternotomy, and pre-CPB (i.e., before CPB initiation). Pre-event values were determined as the average of three NOL index values evenly spaced (i.e., 10 s) and sampled within a 30-s window before the event. Post-event values were determined as the average of three NOL index values evenly spaced (i.e., 10 s) and sampled within a 30-s window starting 60 s after the event. The primary endpoint was the average NOL index over the five sampling time points (NOLav). We considered that NOLav would reflect the nociceptive burden better than comparing groups by single values at each time point. Therefore, the secondary endpoints were the NOL index values and the number of patients with a nociceptive response (i.e., NOL index > 25) at pre-incision, post-incision, pre-sternotomy, post-sternotomy, and pre-CPB.

### 2.4. Statistical Analysis

In the NESP study, we provided a detailed sampling size calculation for the initial primary outcome (i.e., intraoperative fentanyl consumption) that gave a minimum number of patients per group of 28 [18]. For this post hoc study, statistical analyses were performed using NCSS 2022 Statistical Software (NCSS, v22.0.2, LLC, Kaysville, UT, USA). Normality for quantitative data was established with visual inspection and using the Shapiro-Wilk test. Continuous variables were expressed as mean (±SD) or median (interquartile range, IQR, 25th–75th percentiles), depending on their distribution. Comparisons between the two groups were performed with Student’s t-test or the Mann–Whitney U test, as appropriate. Categorical data were presented as category counts and percentages. The χ2 test or Fisher’s exact test was used to check the strength of association between categorical data. The time course of the NOL index values across the five time points was analyzed with a mixed model for repeated measures (MMRM) with random intercepts for patients. The intervention group (i.e., Control vs. ESPB), the time point (i.e., pre-incision, post-incision, pre-sternotomy, post-sternotomy, and pre-CPB), and the interaction between the intervention group and time point were considered fixed effects. All tests were 2-tailed with α set to 0.05. For the MMRM design, the Bonferroni adjusted *p*-value was reported and calculated by multiplying the observed (uncorrected) *p*-value by the number of comparisons made. Hence, the Bonferroni adjusted *p*-value was considered significant if less than 0.05.

## 3. Results

This post hoc analysis included all eighty-three patients from the original NESP randomized controlled clinical trial and two additional ESPB patients initially excluded due to early postoperative bleeding requiring urgent reintervention. Accordingly, we analyzed 43 patients in group 1 (Control) and 42 in group 2 (ESPB).

As previously reported, baseline and perioperative patient characteristics were well-balanced across the study groups (Table 1).

The MMRM analysis of the NOL index time course indicated significant main effects of group (*F*-value = 101.94, *p* < 0.001), time (*F*-value = 42.26, *p* < 0.001), and the interaction of group and time (*F*-value = 23.8, *p* < 0.001).

### 3.1. Primary Outcome

**Figure 2:**
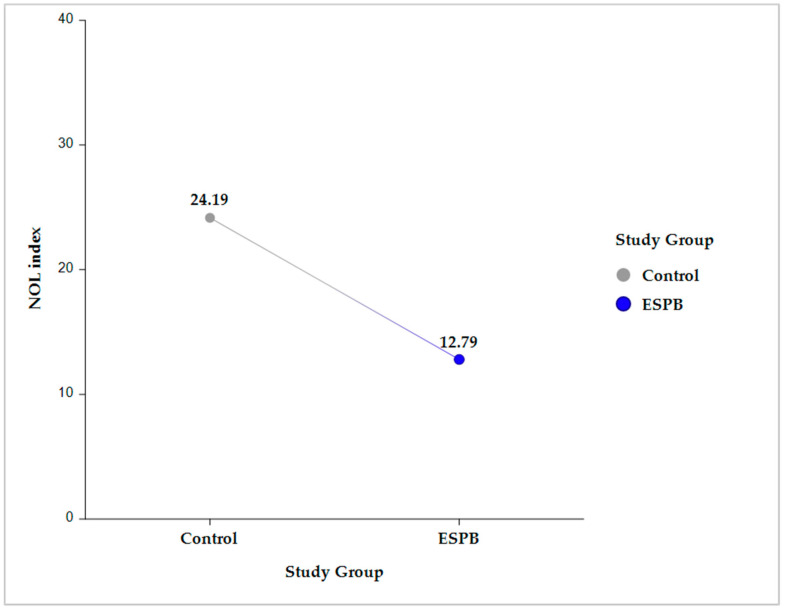
Intergroup comparison (Control vs. ESPB) for the average NOL index value across the five sampling time points. The data are presented as least square adjusted means. For Control vs. ESPB, *p* < 0.001. Analysis was performed using a mixed model for repeated measurement with random intercepts for patients and fixed effects for group, time, and the interaction of group and time. ESPB, erector spinae plane block; NOL, nociception level.

Compared to the control group, the ESPB group had a significantly lower NOLav, 12.78 ± 0.8 vs. 24.18 ± 0.79 (*p* < 0.001) (Figure 2).

### 3.2. Secondary Outcomes

**Figure 3:**
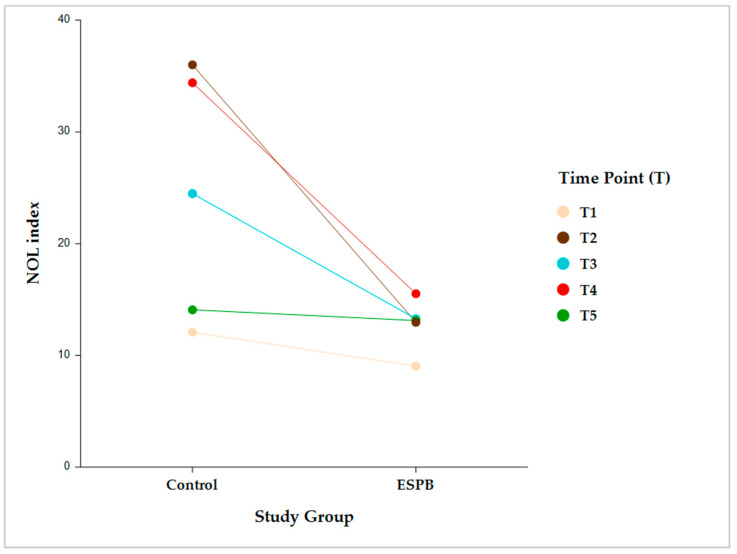
Intergroup comparison (Control vs. ESPB) by time for the NOL index value. The data are presented as least square adjusted means. Time points: T1 (pre-incision), *p* = 0.759; T2 (post-incision), *p* < 0.001; T3 (pre-sternotomy), *p* < 0.001; T4 (post-sternotomy), *p* < 0.001; T5 (pre-CPB), non-significant. Analysis was performed using a mixed model for repeated measurement with random intercepts for patients and fixed effects for group, time, and the interaction of group and time. CPB, cardiopulmonary bypass; ESPB, erector spinae plane block; NOL, nociception level.

Intergroup comparisons showed that, following skin incision, the NOL index was significantly lower in the ESPB compared to the control group at post-incision, pre-sternotomy, and post-sternotomy (*p* < 0.001). However, this difference became statistically insignificant before CPB initiation due to stepwise fentanyl administration according to the prespecified NAN protocol (Figure 3).

**Figure 4:**
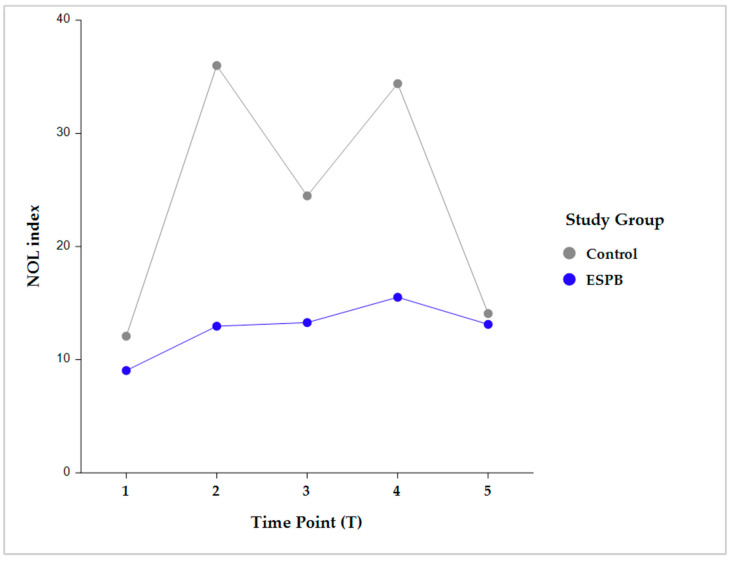
Pairwise intragroup comparison (Control, ESPB) between consecutive NOL index time points. Time point comparisons: pre-incision (T1) vs. post-incision (T2); T2 vs. pre-sternotomy (T3); T3 vs. post-sternotomy (T4); and T4 vs. pre-CPB (T5). The data are presented as least square adjusted means. Control group: *p* < 0.001 for all consecutive pairwise comparisons. ESPB group: lack of statistical significance for all consecutive pairwise comparisons. Analysis was performed using a mixed model for repeated measurement with random intercepts for patients and fixed effects for group, time, and the interaction of group and time. CPB, cardiopulmonary bypass; ESPB, erector spinae plane block; NOL, nociception level.

Intragroup comparisons were performed in each group between two consecutive time points (i.e., pre-incision vs. post-incision; post-incision vs. pre-sternotomy; pre-sternotomy vs. post-sternotomy; and post-sternotomy vs. pre-CPB) (Figure 4).

The NOL index was not significantly different between any two consecutive time points in the ESPB group. Conversely, the control group exhibited significantly different NOL index values between all the consecutive time points (*p* < 0.001) (Table 2).

**Figure 5:**
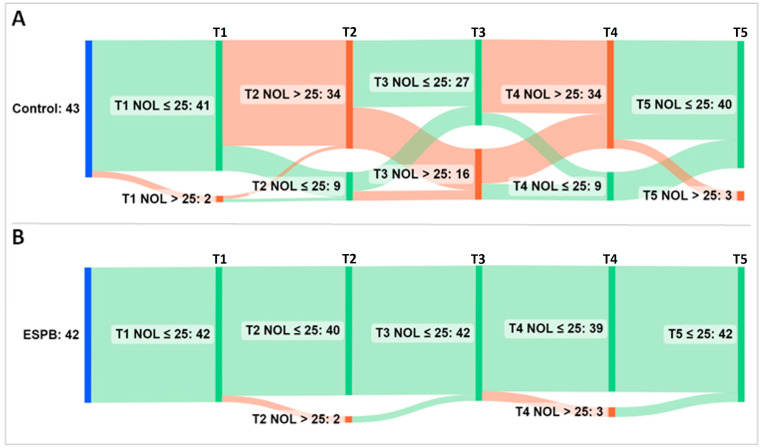
Alluvial diagram showing the flow of patients across five NOL index sampling time points. (**A**) Group Control; (**B**) Group ESPB. The sampling was performed at pre-incision (T1), post-incision (T2), pre-sternotomy (T3), post-sternotomy (T4), and pre-CPB (T5). Time points accompany vertical bars connected by stripes. The cumulative height of the bars per time point is constant within a group and encodes the total number of patients in the group (i.e., 43 patients in the Control and 42 patients in the ESPB group). The height of the stripes encodes the number of patients that shift from one bar to the next. As they leave their source bar, the stripes pick up the color of their target bar. The color of bars and stripes encodes either a non-nociceptive response (i.e., green for NOL index ≤ 25) or a nociceptive response (i.e., red for NOL index > 25) to noxious stimuli. The first bar (blue) encodes patients at baseline. Most of the ESPB patients exhibited a steady NOL index ≤ 25. Conversely, the control group achieved late nociception control at the additional cost of more fentanyl administration than the interventional group. Abbreviations: ESPB, erector spinae plane block; NOL, nociception level.

For further analysis, the NOL index was treated as a categorical variable (i.e., NOL index > 25 representing an inadequate, nociceptive response to noxious stimuli vs. NOL index ≤ 25 representing an adequate, non-nociceptive response to noxious stimuli). An alluvial diagram depicts the flow of patients across the five time points exhibiting nociceptive and non-nociceptive responses (Figure 5).

In addition, the statistical significance for intergroup comparison at each time point is shown in Table 3.

Compared to the controls, significantly fewer ESPB patients reached a NOL index > 25 at post-incision, pre-sternotomy, and post-sternotomy (*p* < 0.001). Patients from both groups with a nociceptive response received fentanyl according to the prespecified NAN protocol, and by CPB initiation, most of them regained a NOL index ≤ 25 (93% Control patients vs. 100% ESPB patients, *p* = 0.241). However, as demonstrated in the original trial, the control group not only achieved nociception control later but did it at the additional cost of more fentanyl consumption compared to the ESPB group (*p* < 0.001) [18].

## 4. Discussion

This post hoc analysis proves that the ESPB group achieved an early and durable control of nociception within a NOL index-directed anesthetic protocol. In contrast, the control patients achieved a late control of nociception at the extra cost of more fentanyl consumption, as demonstrated in the original trial.

Several considerations must be addressed before extracting conclusions. First, a gold standard nociception or pain monitor has yet to be determined. However, multiple NAN surrogates have been introduced in the last decade, including electroencephalography and electromyography, photoplethysmography, heart rate variability, skin conductance, and pupillometry [19]. Unsurprisingly, different monitors likely reflect different NAN states associated with different endocrine stress responses, making comparisons and interchangeability difficult [20]. In addition, the complexity increases with multimodal techniques (i.e., fascial plane blocks, epidurals, non-opioid drugs) and the type of surgery. Notably, cardiac surgery is particularly challenging to monitor for nociception due to CPB and cardiac pacing. The former is expected to render ineffective any photoplethysmographic output, including the NOL index, while the latter was shown to disrupt the surgical photoplethysmographic index [21]. These made compelling arguments to apply the NOL index only until CPB initiation, providing a window of objectivity long enough to assess the block success and fine-tune opioid administration in both study arms. To date, only two other studies monitored nociception intraoperatively in cardiac surgery [22,23]. Interestingly, both used the pupillary pain index and reported reduced sufentanil consumption.

Second, the extent to which nociception monitors can improve clinical practice and outcomes has yet to be determined. A consistent finding among studies comparing nociception-guided anesthesia to standard protocols was an improvement in postoperative pain scores, likely as a result of a better-timed and more personalized intraoperative opioid administration [16,17,24]. Relatedly, our analysis provides objective proof that, compared to ESPB, standard analgesia not only required more opioid consumption but was also asynchronous, lagging behind nociception until after sternotomy. Alternatively, preemptive higher doses of opioid could have circumvented this lag but would have risked overdosing. In contrast, bilateral single-shot ESPB elicited an early, efficient, and sustained preemptive effect that could explain the lower postoperative pain scores beyond the block duration (i.e., up to 48 h after surgery) reported in the original trial [18].

Third, our analysis confirms that nociception monitors lend themselves well to combined regional and general anesthesia. Pinprick and cold stimulation are classically used to assess the extent of sensory block in awake patients, yet this is poorly correlated with intraoperative antinociception [25]. In addition, cutaneous sensory testing becomes impossible in anesthetized patients. However, in both instances, a nociception monitor could prove helpful in evaluating and tracking the block performance. Recently, Ghanty et al. provided anecdotal evidence on using the NOL index during combined general anesthesia and paravertebral blockade [26]. Further studies may explore and compare the analgesic effectiveness of different nerve blocks using NAN monitoring.

Lastly, our study adds to the growing evidence upholding the efficacity of bilateral ESPB in cardiac surgery with midline sternotomy [5,6,7,8]. However, neither this post hoc analysis nor the original trial were equipped to provide mechanistic insight into the ESPB action. Whether and how the local anesthetic spreads anteriorly to achieve parasternal and sympathetic blockade remains unclear because of conflicting anatomical and clinical data. Schwartzamann et al. [27] provided magnetic resonance imaging proof that ESPB shares a similar anesthetic action to paravertebral blocks, hence the label “paravertebral by proxy” [28]. A comprehensive narrative review found twelve out of sixteen cadaveric studies of thoracic ESPB to report paravertebral dye spread, albeit in a minority of cases [25]. Nevertheless, this may be a gross understatement of clinical reality due to several limitations associated with cadaveric studies. Shibata et al. suggested that the paravertebral diffusion of the local anesthetic deposited under the erector spinae muscle might continue for several hours in live subjects, an aspect that escapes dissection studies [29]. In addition, a visual inspection of the dye spread is intrinsically flawed as it invariably underestimates the actual cutaneous sensory loss [25]. On top of that, several anecdotal reports attest to a sympathetic chain block elicited by ESPB [30,31]. Overall, a large body of evidence indicates that bilateral ESPB can achieve an extensive yet variable somatic and visceral blockade.

There are several limitations to our study. First, the post hoc design is inherently biased. Second, given the pragmatic scope of our original trial, we could only retrieve and analyze NOL index data collected at five different time points instead of continuous trends. Indeed, the complete trend analysis may have increased the robustness of data interpretation. Third, the NOL index monitor was applied only until CPB initiation, precluding any objectivity regarding the post-CPB period. Additionally, the NOL index monitor is not a gold standard in itself. Therefore, a second independent loop of nociception control, such as the perioperative levels of stress hormones, could have further endorsed our findings. Finally, the generalizability is limited since the original study was single-centered and had an open-label design. Although sham blocks could have secured a double-blinding design, this practice has remained controversial in several institutions [32], including the authors’.

## 5. Conclusions

This post hoc analysis demonstrates that the addition of bilateral single-shot ESPB to general anesthesia during open heart surgery improved the quality of intraoperative nociception control according to a NOL index-based evaluation.

## Figures and Tables

**Table 1:** Baseline and perioperative patient characteristics.

Parameter	Control (*n* = 43)	ESPB (*n* = 42)
Demographics		
Age, years	63 (55–69)	62 (52–66.25)
Gender, male	26 [60.5]	27 [64.3]
BMI, kg/m^2^	29.6 ± 4.9	28.6 ± 4
Weight, kg	82.8 ± 14.1	83.4 ± 13.9
Surgery characteristics		
Duration of surgery, minutes	296.8 ± 64.1	283.3 ± 59
CPB time, minutes	95 (82–106)	90 (68.75–110.5)
Aortic cross-clamp time, minutes	66 (56–82)	58 (43.5–79.75)
Surgical procedures		
CABG (±valve surgery)	22 [51.1]	20 [47.6]
Valve surgery	17 [39.5]	17 [40.5]
Miscellaneous	4 [9.3]	5 [11.9]
Medication history		
RAAS inhibitors	28 [65.1]	26 [61.9]
Beta blockers	34 [79]	36 [85.7]
Anticoagulants	1 [2.3]	4 [9.5]
Antiplatelet drugs	29 [67.4]	29 [69]
Chronic opioid therapy	0 [0]	0 [0]
Preoperative risk		
EuroSCORE II	1.07 (0.76–1.39)	1.01 (0.85–1.55)
Intraoperative monitoring ^‡^		
Heart rate, bpm	84 (75–90)	88 (79.25–90)
Mean arterial pressure, mmHg	67.2 ± 8.1	65.9 ± 7.6
Lactate, mmol/L	1.4 (1.1–1.6)	1.3 (1–1.7)
BIS	45 (41–47)	43 (41–48)

Values are mean ± SD, median (IQR, 25th–75th percentiles), or number [percentages]. There were no intergroup differences for any parameter. ^‡^ measurements were recorded before cardiopulmonary bypass cannulation. Abbreviations: BIS, Bispectral index; BMI, body mass index; CABG, coronary artery bypass graft; CPB, cardiopulmonary bypass; ESPB, erector spinae plane block; EuroSCORE, European System for Cardiac Operative Risk Evaluation; IQR, interquartile range; RAAS, renin-angiotensin-aldosterone system; SD, standard deviation.

**Table 2:** Intergroup and intragroup comparison of the NOL index time course.

Time Point	NOL Index Value		Intergroup Comparison (*p-*Value) Control vs. ESPB	Intragroup Comparison (*p-*Value)	
	Control	ESPB		Control	ESPB
Tav	24.18 ± 0.79 (22.6–25.76)	12.78 ± 0.8 (11.18–14.38)	<0.001	N/A	N/A
T1	12.07 ± 1.47 (9.18–14.97)	9.07 ± 1.49 (6.14–12)	0.759	N/A	N/A
T2	35.97 ± 1.47 (33.08–38.87)	12.95 ± 1.49 (10.02–15.88)	<0.001	T1 vs. T2: <0.001	T1 vs. T2: NS
T3	24.44 ± 1.47 (21.54–27.33)	13.28 ± 1.49 (10.35–16.21)	<0.001	T2 vs. T3: <0.001	T2 vs. T3: NS
T4	34.39 ± 1.47 (31.5–37.29)	15.52 ± 1.49 (12.59–18.45)	<0.001	T3 vs. T4: <0.001	T3 vs. T4: NS
T5	14.04 ± 1.47 (11.15–16.94)	13.09 ± 1.49 (10.16–16.02)	NS	T4 vs. T5: <0.001	T4 vs. T5: NS

Values are least square adjusted (LSA) means ± SE and 95% confidence interval (CI) for LSA means. The intergroup comparison shows that the NOL index was significantly lower in the ESPB-to-Control group as an average over the five sampling time points (Tav) and at post-incision (T2), pre-sternotomy (T3), and post-sternotomy (T4) but not at pre-incision (T1) and pre-CPB (T5). The intragroup pairwise comparison between consecutive time points reflects a fluctuating response to nociception in the control group compared to the ESPB group. Analysis was performed using a mixed model for repeated measurements (MMRM) with random intercepts for patients. MMRM indicated main effects of group (*p* < 0.001), time (*p* < 0.001), and the interaction of group and time (*p* < 0.001). Abbreviations: CPB, cardiopulmonary bypass; ESPB, erector spinae plane block; N/A, non-applicable; NOL, nociception level; NS, non-significant; SE, standard error of mean.

**Table 3:** Intergroup comparison of number of patients with nociceptive response.

	No. of Pts. with NOL Index > 25		Intergroup Comparison (*p-*Value) Control vs. ESPB
	Control (*n* = 43)	ESPB (*n* = 42)	
T1	2 [4.6]	0 [0]	0.494
T2	34 [79]	2 [4.7]	<0.001
T3	16 [37.2]	0 [0]	<0.001
T4	34 [79]	3 [7.1]	<0.001
T5	3 [7]	0 [0]	0.241

Values are number [percentages]. A nociceptive response to noxious stimuli is considered a NOL index > 25. Analysis was performed using the χ2 test or Fisher’s exact test, as appropriate. Abbreviations: ESPB, erector spinae plane block; NOL, nociception level; T1, pre-incision; T2, post-incision; T3, pre-sternotomy; T4, post-sternotomy; T5, pre-CPB.

## Data Availability

Data used in this study may be provided by the corresponding author upon reasonable request.
